# Self-perceived functional impairment or disability in Delusional Disorder – Case Reports

**DOI:** 10.1192/j.eurpsy.2022.1977

**Published:** 2022-09-01

**Authors:** M.D.R.D.R.F.D. Basto, A. Certo, O. Nombora, A. Horta, E. Mendes, Â. Venâncio

**Affiliations:** 1 Centro Hospitalar de Vila Nova de Gaia e Espinho, Serviço De Psiquiatria E Saúde Mental, Vila Nova de Gaia, Portugal; 2 Vila Nova de Gaia Hospital Center, Psychiatry And Mental Health Service, Vila Nova de Gaia, Portugal

**Keywords:** Delusional disorder, Psychosis, cognitive deterioration

## Abstract

**Introduction:**

Delusional disorder (DD) is a psychotic disorder with an estimated prevalence of less than one percent,traditionally characterized by systematized delusional ideas with no cognitive deterioration.However, some studies have been reporting impairment of neurocognitive system (social cognition,learning and memory, expressive language,complex attention, executive function) that might have an impact functionality both in social and work domains.^.^

**Objectives:**

This work aims to review clinical evidence on self-perceived functional impairment or disability in DD and to present two clinical cases evaluated at a psychiatric unit.

**Methods:**

We report two clinical cases based on patients’ history and clinical data, and reviewed clinical records using PubMed® database with search terms of “Delusional Disorder”,“Cognition Impairment in Persistent DD”.

**Results:**

We present two clinical cases of patients who were admitted to psychiatric unit after developing psychotic symptoms namely persecutory delusions about neighbors.A persistent delusional disorder was established and antipsychotic treatment was initiated.The 74-years-old men presented deficits in executive and memory processes; ended up institutionalized after two months of being discharged.The 47-years-old woman, despite remaining as a lawyer, noticed a decrease in work capacity and so she ended up being responsible for less demanding cases.Cases of delusional disorder showed a poor performance in most cognitive tests and some of the cognitive deficits seem to affect functionality namely memory, expressive language and attention.

**Conclusions:**

Although classical literature has not systematized an association between DD and personality deterioration,there are some evidences of loss of functionality and cognitive commitment in this disorder.This suggests the importance of cognitive interventions to improve functional prognosis in this clinical population.

**Disclosure:**

No significant relationships.

